# Comparison between titanium mesh and autogenous iliac bone graft to restore vertebral height through posterior approach for the treatment of thoracic and lumbar spinal tuberculosis

**DOI:** 10.1371/journal.pone.0175567

**Published:** 2017-04-13

**Authors:** Yongjian Gao, Yunsheng Ou, Qianxing Deng, Bin He, Xing Du, Jianxiao Li

**Affiliations:** 1 Department of Orthopedics, the First Affiliated Hospital of Chongqing Medical University, Chongqing, P.R. China; 2 Department of Orthopedics, the Fengdu people’s Hospital of Chongqing, Chongqing, P.R. China; Harvard Medical School/BIDMC, UNITED STATES

## Abstract

**Object:**

To compare the clinical efficacy of titanium mesh cages and autogenous iliac bone graft to restore vertebral height through posterior approach in patients with thoracic and lumbar spinal tuberculosis.

**Method:**

59 patients with spinal tuberculosis underwent interbody fusion and internal fixation through posterior approach in our department from January 2011 to December 2013. In group A, 34 patients obtained titanium mesh for the reconstruction of vertebral height, among them 25 patients (group A1) suffered from single-segment spinal tuberculosis, and 9 patients, (group A2) had multi-segment spinal tuberculosis. In group B, 25 patients got autogenous iliac bone graft to restore vertebral height, including 24 patients with single-segment spinal tuberculosis (group B1), and 1 patient with multi-segment spinal tuberculosis (group B2). The clinical efficacy was evaluated based on average operation time, blood loss, hospital stays, hospitalization expenses, visual analog scale (VAS), Oswestry Disability Index (ODI), erythrocyte sedimentation rate (ESR), C-Reactive protein (CRP), neurological function recovery, bony fusion, intervertebral height, Cobb angle and postoperative complications.

**Results:**

Final follow-up time was an average of 35.5 months ranging from 15 to 56 months. All patients were completely cured and obtained solid bone fusion. The bony fusion time was 9.4±6.1 months in group A1, 10.2±2.7 months in group A2 and 8.7±3.6 months in group B1. There were no significant difference among three groups (*P*>0.05). The Cobb correction and restoration of intervertebral height significantly improved compared with those in preoperation, but without significant difference among three groups (*P*>0.05). The loss of angular correction and intervertebral height in group A1 were found to be less than those in group B1 (P<0.05), but with no significant difference between group A1 and group A2, and between group A2 and group B1 (P>0.05). Patients in group B1 got the most loss of angular correction and intervertebral height. In addition, neurological function was revealed to be significantly improved after surgery. There were significant differences of VAS, ODI, ESR and CRP between preoperation and postoperation at the final follow-up time (*P*<0.05), with no significant difference among three groups (*P*>0.05). No statistically significant difference was found when analyzing blood loss, hospital stays, hospitalization expenses, and corrective cost among three groups (*P*>0.05). Complications included cerebrospinal fluid leakage (2 cases in group A1 and group A2), sinus formation (3 cases in group A1, group A2 and group B1), and intervertebral infection (1 case in group B1), but no implant failure or donor site complications was found in any patient.

**Conclusions:**

Titanium mesh cages could obtain good clinical efficacy comparable to autogenous iliac bone graft when treating single-segment spinal tuberculosis, and may be better than autogenous iliac bone graft for treating multi-segment spinal tuberculosis.

## Background

Tuberculosis has become increasingly widespread in our country [1, 2]. Bone and Joint tuberculosis (BJTB) constitutes about 10% of total extra-pulmonary TB cases[3], and spinal tuberculosis is known as the most commonly infected site among skeletal tuberculosis (about 44%) [4]. The intervertebral disc and the end plates of the adjacent superior and inferior vertebral bodies are often involved in spinal tuberculosis, and their severe destruction can result in kyphotic deformity and even paraplegia[5]. Various studies have shown that the majority (82–95%) of spinal tuberculosis patients obtain good clinical outcome after receiving current chemotherapy. Surgical intervention is required if the patients suffer from neurologic deficit, big abscess formation, persistent or recurrent infection, severe pain, local kyphosis, and segmental instability[1, 2, 5–7].

Surgical intervention has become an important way of treating serious spinal tuberculosis, and the anterior-posterior approach is known as the gold standard for surgery, but limited by severe trauma, great blood loss, and high risk [2]. Many studies have revealed that posterior approach is able to achieve good clinical outcome when treating spinal tuberculosis [1, 2, 6, 8, 9]. Vertebral height demands reconstruction after surgical debridement, but it is still elusive to select appropriate bone-grafting materials to restore vertebral height [1, 10–13]. In clinical medicine, titanium mesh and autogenous iliac bone graft are ubiquitous. This study is to compare the efficacy between titanium mesh cages and autogenous iliac bone graft to restore vertebral height through posterior approach in patients with spinal tuberculosis.

## 1 Materials and methods

### 1.1 Ethics statement

This study was approved by the Institutional Review Board of the First Affiliated Hospital of Chongqing Medical University and conducted according to the principles of the Declaration of Helsinki. All of the participants provided their written informed consent to participate in this study, before their data were stored in the hospital database and used for research purposes.

### 1.2 Patient population

Inclusion criteria: thoracic and lumbar spinal tuberculosis in adults, posterior approach, internal fixation, and reconstruction using titanium mesh cages or autogenous iliac bone graft. Exclusion criteria: active pulmonary tuberculosis and extrapulmonary tuberculosis, cancer, discontinuous spinal tuberculosis, osteoporosis, traumatic fractures, thoracic and lumbar surgery within 6 months.

From January 2011 to December 2013 in our department, there were 147 patients with thoracic and lumbar tuberculosis, but 59 patients were included in this study. Of the 59 patients, 25 cases (42.4%) had typical symptoms of tuberculosis, including night fever, loss of weight, fatigue, and back pain. Diagnosis was based on non-specific laboratory and imaging findings, including spinal radiographic films, computed tomography (CT) and magnetic resonance imaging (MRI) in order to verify vertebral body destruction or collapse, intervertebral space narrowing or disappearing and cold abscess. Spinal tuberculosis was examined by postoperative pathological analysis in all patients. 34 cases of 59 patients with spinal tuberculosis in group A obtained titanium mesh cages for reconstruction involving 25 patients with single-segment spinal tuberculosis (group A1) and 9 patients with multi-segment spinal tuberculosis (group A2). In group B, 25 cases got autogenous iliac bone graft for reconstruction including 9 patients with single-segment spinal tuberculosis (group B1) and 1 patients with multi-segment spinal tuberculosis (group B2, 70 years old and T6-8). No significant difference of general datas was found among three groups (P>0.05, Table 1).

**Table 1:** General data of three groups.

Items	Group A1	Group A2	Group B1
Age(Y)	39.2±14.2	42.4±12.5	41.4±14.3
Sex (M/F)	13/12	7/2	9/15
Thoracic	3	4	3
Thoracolumbar	8	3	7
Lumbar	14	2	14
Para-vertebral abscess	10	7	14
Psoas abscess	6	2	7
Iliac fossa abscess	3	1	3

Age was expressed as mean±SD, and t-test was performed; P = 0.974. M: male; F: female; n: number of patients.

### 1.3 Preoperative management

All people had to achieve chemotherapy shortly after clinical diagnosis was suspected. Anti- tuberculosis drugs with HREZ chemotherapy regimen consistingof isoniazid (300 mg/day), rifampicin (450 mg/day), ethambutol (750 mg/day), and pyrazinamide (1500 mg/day) were administered 2–4 weeks before surgery. Surgical management was performed when ESR decreased below 60 mm/h and CRP was progressively decreased..

### 1.4 Surgical management

In group A, patients were placed in the prone position after administering general endotracheal anesthesia. A posterior midline incision was made following the focus of segmental lesions, and paraspinal muscles were peeled along the debridement side. Wiltse approach was applied on the non-debridement side. Transpedicular screws were fixed in two levels superior and inferior to the level of decompression, and the screws were also placed in the affected vertebrae if the upper part of the vertebrae and pedicle was not involved. A temporary pre-bent rod was installed on the non-debridement side to achieve spine stability. We preferred radical debridement. Streptomycin 1.0 g and isoniazid 0.2 g were administered locally after preparing bone graft bed. The kyphosis was slowly and carefully corrected with the help of the compression and stretching of the internal fixation instrumentation. The specially formed titanium mesh cages (filled with resected cancellous bone containing Streptomycin 1.0 g) were inserted into the bone trough, and had complete connection with upper and lower vertebral end-plates. Negative pressure drainage and incision sutures were performed. In group B, surgical methods were same as group A. After preparing bone graft bed, autogenous iliac was clipped to fit the vertebral defect.

Abscess treatment: For the para-vertebral and psoas abscess, abscess cavity wall, and caseous necrosis and tubercular granulation tissue were eliminated. Large psoas and iliac fossa abscess underwent debridement by extraperitoneal approach, and Streptomycin 1.0 *g* was used in the lesion site. Effective drainage with a flushing piping and a drainage tube system was applied after debridement. And resected specimens were collected for bacterial culture and pathological diagnosis.

### 1.5 Postoperative care

The drainage tube was pulled out when drainage volume was less 30 ml/day. Patients obtained oral HREZ chemotherapy postoperatively. 12 months later, pyrazinamide was discontinued. Patients received 18- to 24- month regimens of HRE chemotherapy (12HREZ/18-24HRE). Ambulation was allowed four weeks after surgery. All patients were examined clinically and radiologically in 1 week, and in 3, 6, 12, 18 and 24 months after surgery, and then once a year.

### 1.6 Evaluating standard

#### 1.6.1 Clinical assessments

For all cases, the following indexes were recorded preoperatively, three months of postoperation, and at follow-up time: (1) average operation time, blood loss, hospital stays, hospitalization expenses, Visual Analogue Scale (VAS), improvement in the postoperative Oswestry Disability Index (ODI) score, (2) recovery of neurologic function, as assessed by the Frankel grade, (3) laboratory tests, including erythrocyte sedimentation rate (ESR) and C-reactive protein (CRP).

#### 1.6.2 Radiological assessments

Intervertebral height, kyphotic angle and Cobb angle were recorded. Intervertebral height was defined as the vertical height between superior and inferior vertebral body in coronal plane with fusion zone[14]. Bone grafting fusion was assessed using the radiologic criteria of Bridwell et al[15].

### 1.7 Statistical analysis

All statistical analyses were performed with SPSS version 22.0 statistical software (SPSS, Inc., Chicago, IL, USA). The paired t test was used to analyze data in each group, and independent sample t-test was performed between groups. Values of P < 0.05 were considered statistically significant.

## 2. Results

### 2.1 Clinical assessments

Final follow-up time was an average of 35.5 months (15 to 56 months). VAS, ODI, ESR and CRP were obviously reduced at the final follow-up time than postoperation (P<0.05). The operation time in group A2 was significantly longer than other groups (P < 0.05), but there were no significant difference between group A1 and group B1(P > 0.05, Table 2). 5 patients in group A1, 4 patients in group A2 and 5 patients in group B1 were rated as D grade of neurological function at preoperation, and all patients was improved after surgery apart from 1 case in group B1 who was rated as grade D at the final follow-up.

**Table 2:** Comparison of VAS, ODI, ESR, CRP and operation time, blood loss, hospital stay, and hospitalization expenses pre- and postoperatively in and among three groups (X± S).

	Group A1	Group A2	Group B1	P_A1-A2_	P_A1-B1_	P_A2-B1_
follow-up time	35.8±11.5	36.9±6.7	34.8±7.0	0.791	0.700	0.434
Preoperative VAS	5.9±1.3	5.8±1.5	5.7±1.3	0.844	0.560	0.832
Follow-up VAS	1.7±1.4*	1.8±1.3*	1.1±0.8*	0.915	0.055	0.163
Preoperative ODI	64.9±15.3	69.2±15.2	60.8±12.1	0.470	0.307	0.108
Follow-up ODI	12.8±9.1*	13.6±7.5*	13.9±11.4*	0.826	0.728	0.947
Preoperative ESR	41.5±27.8	60.8±67.2	43.9±26.1	0.224	0.761	0.261
At 3 months postoperative ESR	13.0±10.6*	11.1±4.1*	13.4±9.1*	0.620	0.889	0.489
Preoperative CRP	24.5±18.2	34.8±26.0	30.1±19.3	0.206	0.308	0.573
At 3 months postoperative CRP	10.3±4.4*	8.9±5.3*	14.3±10.4*	0.439	0.100	0.161
Operation time (min)	240.0±52.5	320±77.4#	240.8±66.2	0.016	0.961	0.006
Blood loss (ml)	728.0±505.0	944.4±600.2	518.8±303.2	0.302	0.085	0.071
Hospital stay (days)	18.7±9.6	20.8±6.4	17.9±10.1	0.550	0.776	0.428
hospitalization expenses	73006.0±18860.1	66712.2±8618.3	76216.5±20894.0	0.194	0.575	0.074

*, ^#^ Significant difference at P<0.05 compared with pre-operation and group A1, respectively.

### 2.2 Radiological assessments

**Fig 1:**
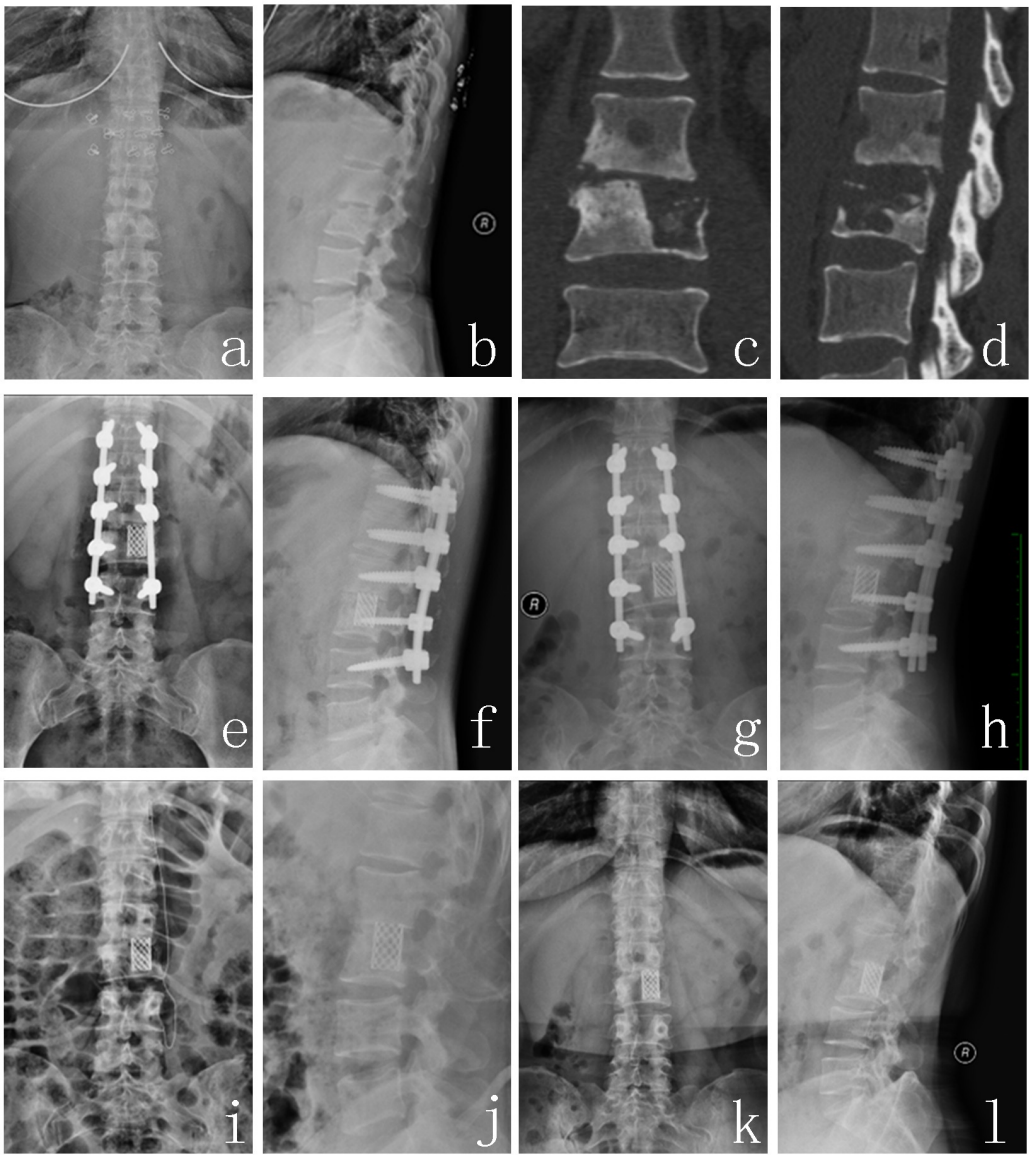
A 36-year-old female patient with L2-3 spinal tuberculosis (Frankel grade E) obtained formed titanium mesh for reconstruction. (a~d) Preoperative X-ray and CT showed that L2 and L3 vertebra body and the intervertebral disc were destroyed, and lumbar instability was formed. (e, f) Postoperative X-ray. (g, h) X-ray at 36 months after surgery. (i, j) Removing the internal fixation at 36 month postoperatively. (k, l) X-ray of 1 year after implant removal showed good location of titanium mesh and lumbar physiological curve.

**Fig 2:**
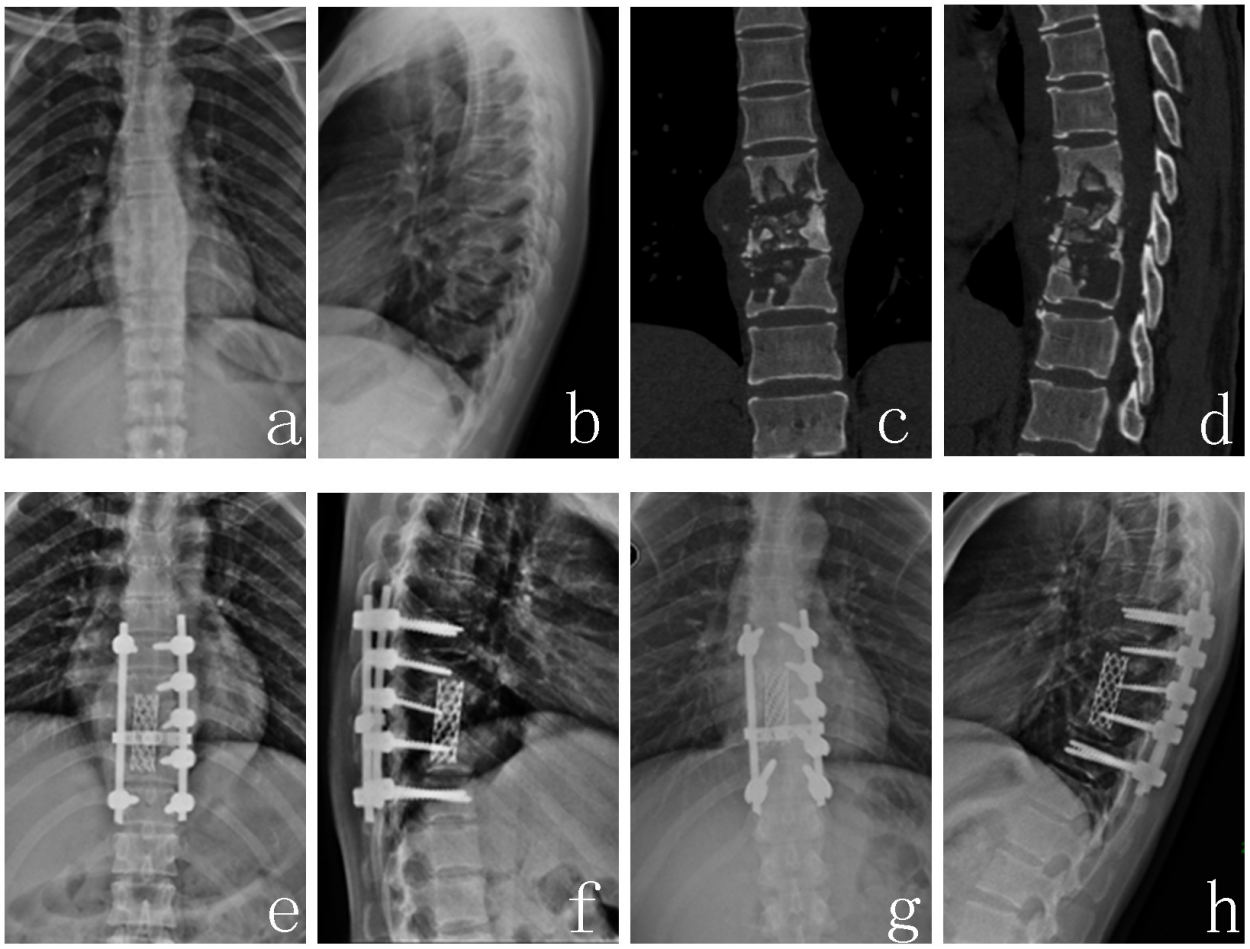
A 41-year-old female patient with T8,9,10 spinal tuberculosis (Frankel grade E) got formed titanium mesh for reconstruction. (a~d) Preoperative X-ray and CT demonstrated that T8-10 vertebra body and the intervertebral discs were destroyed and instability was formed. (e, f) Postoperative X-ray, (g, h) A-P and lateral X-ray at 31 months after surgery revealed good location of titanium mesh and lumbar physiological curve.

**Fig 3:**
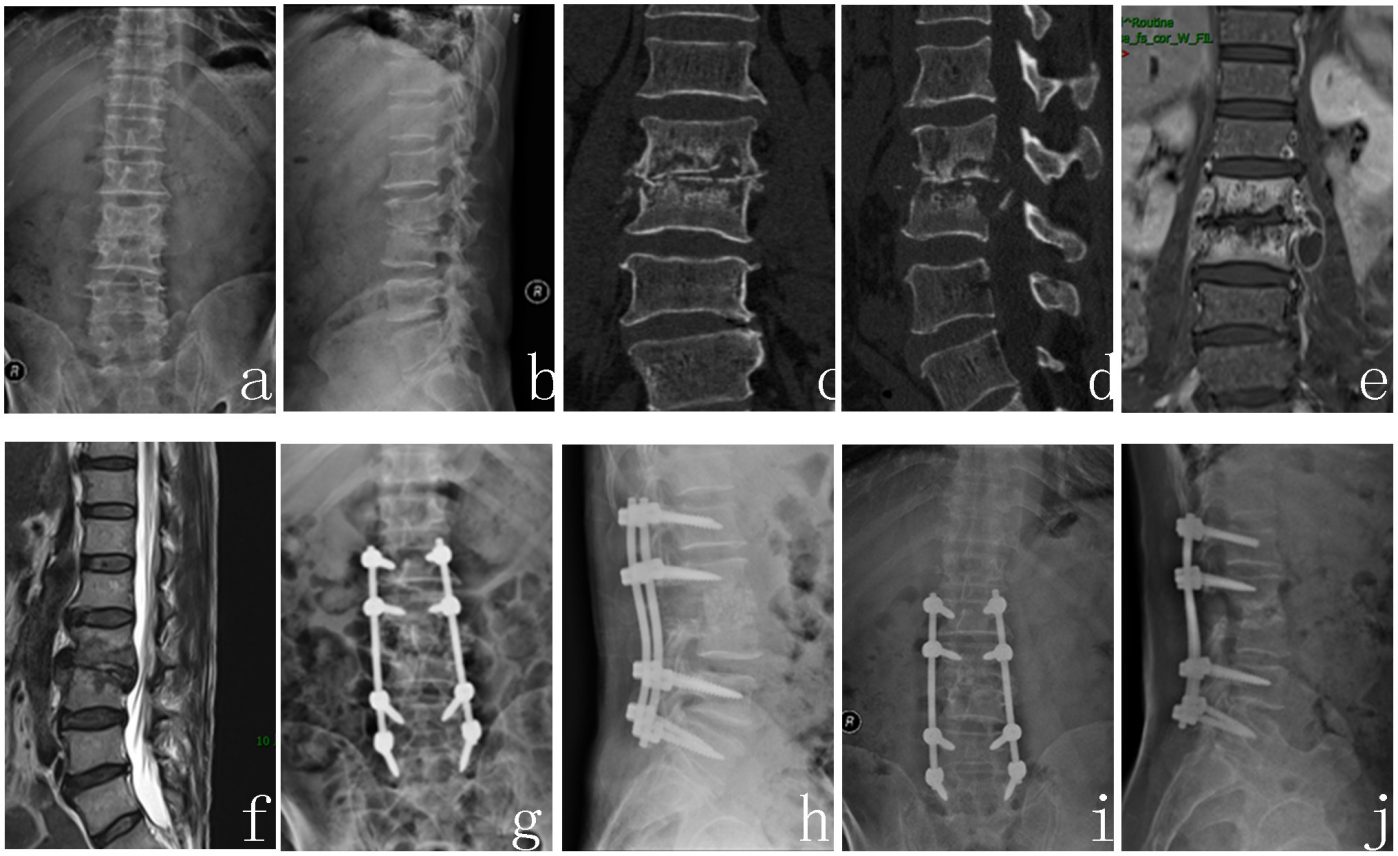
A 58-year-old male patient with L2-3 spinal tuberculosis (Frankel grade D) achieved autogenous iliac bone graft for reconstruction. (a~f) Preoperative X-ray, CT, and MRI showed that L2 and L3 vertebra body and the intervertebral disc were destroyed to produce neurological compression and instability. (g, h) Postoperative X-ray. (I, j) A-P and lateral X-ray at 29 months after surgery showed good location of titanium mesh, lumbar physiological curve and neurologic function recovery.

Spinal tuberculosis was completely cured, and the solid bony fusion was achieved for all patients. The bony fusion time was 9.4±6.1 months in group A1 (Fig 1), 10.2±2.7 months in group A2 (Fig 2), 8.7±3.6 months in group B1 (Fig 3). There was no significant difference among three groups (P>0.05). Kyphosis correction was (4.4±8.3) °, (2.3±32.7) ° and (5.4±12.5) ° in group A1, A2 and B1 respectively. And their restoration of intervertebral heights was 1.8±1.1, 2.3±1.4, 1.8±1.1 cm, respectively. There was also no significant difference among three groups (P>0.05). The Cobb angle loss and intervertebral height loss in group A1 were less than that in group B1(P<0.05), but no significant difference was found between group A1 and group A2, and between group A2 and group B1 (P>0.05), and their loss in group B1 was the greatest in the three groups (Table 3).

**Table 3:** All radiological parameters (X ± s).

	Group A1	Group A2	Group B1	P_A1-A2_	P_A1-B1_	P_A2-B1_
Preoperative kyphosis(°)	20.3±18.3	32.1±17.8	24.5±15.3	0.795	0.722	0.985
Postoperative kyphosis(°)	24.5±17.7*	21.8±18.7	23.8±17.5	0.589	0.819	0.713
Follow-up kyphosis(°)	25.6±17.8*&	24.1±18.5&	26.3±17.6&	0.582	0.885	0.675
kyphosis correction(°)	4.4±8.3	2.3±32.7	5.4±12.5	0.851	0.748	0.788
Loss of correction (°)	1.1±1.8*	2.3±0.8*	2.5±2.0*#	0.054	0.012	0.796
Preoperative intervertebral height(cm)	11.9±2.4	15.4±3.0	12.3±1.8	0.001	0.444	0.001
Postoperative intervertebral height(cm)	13.7±2.6*	17.7±3.8*	14.2±2.0*	0.001	0.478	0.024
Follow-up intervertebral height (cm)	12.9±2.5*&	16.4±4.0*&	12.0±2.2*&	0.027	0.018	0.010
intervertebral height correction (cm)	1.8±1.1	2.3±1.4	1.8±1.1	0.307	0.978	0.336
Loss of intervertebral height(cm)	0.8±0.6*	1.3±1.0*	2.2±1.2*#	0.105	0.000	0.055
Bone fusion(cm)	9.4±6.1	10.2±2.7	8.7±3.6	0.714	0.594	0.254

*, ^&^, ^#^ Significant difference at P<0.05 compared with pre-operation, post-operation, and group A1, respectively.

### 2.3 Complications

No implant failure or donor site complications were found in any patient. There were 20 kinds of complications in 15 patients, such as intervertebral infection, sinus formation, combined bacterial infection, liver and kidney dysfunction. Two cases suffered from cerebrospinal fluid leakage. 1 case in group A1 had tuberculosis meningitis after 1 year (Table 4).

**Table 4:** Complications.

Postoperative Complications	In total	Group A1	Group A2	Group B1
Cerebrospinal Fluid Leakage	2	1	1	0
Drug-induced liver Dysfunction	4	2	0	2
Drug-induced Kidney Dysfunction	7	4	1	2
Postoperative Infection	7	3	2	2
Sinus Formation	3	1	1	1
Bacterial Meningitis	2	1	1	0
Intervertebral Infection	1	0	0	1
Tuberculous Meningitis	1	1	0	0
In total	20	10	4	6

### 2.4. Typical case analysis (Figs 1, 2, 3)

## 3 Discussions

Tuberculosis has become increasingly widespread in china. Various studies have shown that the majority (82–95%) of spinal tuberculosis patients resulted in good clinical outcome after current chemotherapy[16]. Duration of antituberculous treatment remained controversial. Existing guidelines recommended the treatment duration ranging from 6 to 24 months. Van Loenhout-Rooyackers et al.[17] suggested that 6-month antituberculous treatment is probably sufficient. World Health Organization recommends 9 months of treatment for patients with osteoarticular tuberculosis[18]. The American Thoracic Society recommends 6 months of chemotherapy for spinal tuberculosis in adults and 12 months in children[19]. The British Thoracic Society recommends 6 months of daily treatment (2HREZ/4HR), irrespective of age[20]. Due to the serious risk of disability and the difficulty in assessing treatment response, many experts still preferred 12–24 months of antituberculous treatment based on radiological change and inflammatory markers [16, 17, 21]. Duration of antituberculous treatment in China varied from 15 to 36 months (6-12HREZ/9-24HRE)[1, 6, 22–25]. Clinic Society of Chinese Antituberculosis Association doesn’t recommend a short period chemotherapy for patients after surgery[26]. Certainly, anti-tuberculosis drugs should be changed when encountering multidrug-resistant TB.

Preoperative managements are very important for perioperative period care. The ESR and CRP are of a high level, which represent that the tuberculosis bacillus are active and TB toxicity symptoms are serious. ESR and CRP can’t return to normal within 2–4 weeks after chemotherapy, because of abscesses persisting, but TB toxicity symptoms may be relieved. When ESR decreased below 40–80 mm/h or returned to normal, and CRP was progressively decreased[22, 24, 27, 28], the surgery was recommended. In addition, if the patients encountered neural function aggravation, defecation function disturbance, and paraplegia, the surgical intervention should be perfomed immediately.

Surgical treatment methods for spinal tuberculosis mainly included anterior approach, staged or simultaneous anterior decompression combined with posterior stabilization, as well as posterior approach. Anterior approach allowed direct access to the lesion site and was beneficial to debridement and convenient bone grafting[29], but with high risk of pseudarthrosis and ineffective correction of kyphosis. Moreover, the anterior exposure of the upper thoracic spinal region showed some difficulty because of the barrier of thoracic bones, clavicle, costal bone and superior mediastinum organs[8, 29–31]. Anterior approach was limited by long operation time, large amount of bleeding, large wounds, and prolonged bed rest[32]. With the development of pedicle screws and bone fusion, posterior approach had become widespread and benefited to correction of kyphosis and spinal stability [1, 6, 31]. Our research showed that the posterior approach allowed the sufficient decompression of spinal cord and nerve, and bone fusion and good clinical outcome were available using titanium mesh cages or autogenous iliac bone graft combined with fixation instrumentation. Kyphosis correction effect was obviously improved and maintained at the final follow-up. The solid bony fusion was achieved for all patients. Fewer complications related to operation occurred than anterior approach [6, 7, 10].

According to the three column theory of Denis[33], it’s very important to integrate anterior column and middle column for improving spinal stability. Spinal tuberculosis often destroyed spinal anterior column and middle column to reduce stability. Autogenous iliac bone graft was known as the gold standard to repair bone defects due to good osteogenesis, bone induction, bone conductibility and biocompatibility [10]. Recently, many studies report that titanium mesh cages showed an important potential in reliable spinal reconstruction, high bone fusion, sufficient sagittal profile maintenance and low implant-related problems[6, 10, 34, 35].

In this research, the single-segment titanium mesh group and autogenous iliac bone group achieved solid bone fusion, and neurological function in all patients was obviously improved after surgery apart from 1 case in group B1 who was rated as grade D at the final follow-up. Cobb angle loss and intervertebral height loss in group A1 were less when compared with that in group B1. Our results were consistent with previously published results[2, 5, 34]. On the one hand, titanium mesh cages allowed tailored design of shape corresponding to individual bone defects. Titanium mesh cages was filled with resected cancellous bone, which consisted of excised vertebral lamina and articular process, and the donor site complications were avoided. And its insertion to the resection site provided a large interbody—bone interface beneficial to improve spinal stability. Posterior instrumentation and titanium mesh cages were used in our patients to enforce operative segments stability, induce deformity correction and maintenance, bone fusion and prevention of bone resorption. Cobb angle loss and intervertebral height loss using autogenous iliac bone graft were revealed to be greater than that using titanium mesh cages, which may be mediated by bone resorption. In the current study, solid bony fusion, good clinical outcomes as well as improvement of neurologic function were achieved in single-segment titanium mesh group and autogenous iliac bone group, and a small loss of kyphosis and intervertebral height correction was acceptable[7].

The autogenous iliac bone graft showed the risk of stress fractures, fixation failure and severe loss of correction of kyphosis when treating multi-segment spinal tuberculosis which demanded bone grafts with strong mechanical force for spinal stability [29]. The donor site complications included chronic pain (up to 40% of cases) and infection [34, 36]. Zeng et al[1] and Korovessis et al[12] reported that a long-segment bone graft was more prone to result in delayed stress fractures, poor correction and implant failure. In previous studies[13, 25, 29, 37, 38] and our research, titanium mesh was found to provide better structural support, solid bony fusion, kyphosis and intervertebral height correction, the smaller loss of angular correction and intervertebral height than autogenous iliac bone when treating multi-segment spinal tuberculosis. Bone tablets grafts harvested from intraoperative biting were sufficient to fill the titanium mesh for stimulating bone fusion.

One patient in group A1 suffered from tuberculosis meningitis 1 year post-operation.. Drug-induced liver and kidney dysfunction, tuberculosis and bacterial meningitis, intervertebral infection, sinus formation and combined staphylococcus aureus were cured by debridement, antibiotics and the symptomatic methods. That complications may be related with irregular anti-TB drugs treatments, drug-resistance, malnutrition, poor immunity and so on.

## 4 Conclusion

Titanium mesh cages was found to be a good alternative to autogenous iliac bone graft for restoring vertebral height when treating single-segment spinal tuberculosis and might obtained better clinical efficacy than autogenous iliac bone graft for treating multi-segment spinal tuberculosis. Some potential shortcomings of our study should be considered. Many studies involve anterior and posterior multi-segment autogenous iliac bone and titanium mesh bone graft, and combined anterior and posterior approaches are necessary. Further, a larger number of patients and longer follow-ups would be required.

## Supporting information

S1 File Ethical review. It contains the Ethical Approval Form and translation document.
(ZIP)

S2 File Informed consent form template for clinical trials. It’s a informed consent form template for clinical trials.
(ZIP)
