# Supplementary figures and images for: Comparison between titanium mesh and autogenous iliac bone graft to restore vertebral height through posterior approach for the treatment of thoracic and lumbar spinal tuberculosis
Source: PLoS One. 2017 Apr 13;12(4):e0175567. doi: 10.1371/journal.pone.0175567 (PMC5391077; doi:10.1371/journal.pone.0175567)

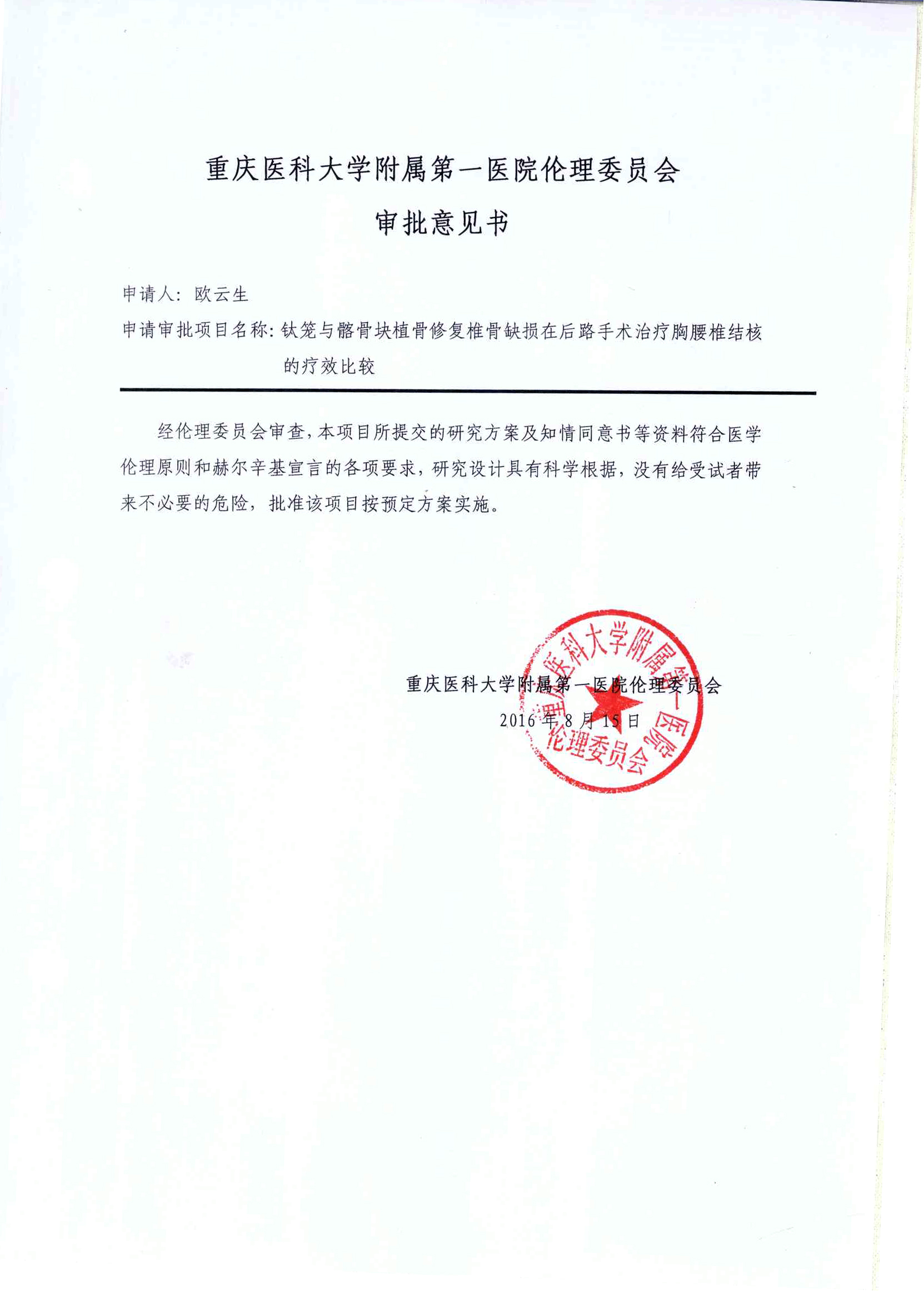

Supplement: S1 File — It contains the Ethical Approval Form and translation document. (ZIP) [file pone.0175567.s001.zip › Ethical Review/Ethical Review.jpg]
