# Supplementary material for: Comparison between titanium mesh and autogenous iliac bone graft to restore vertebral height through posterior approach for the treatment of thoracic and lumbar spinal tuberculosis
Source: PLoS One. 2017 Apr 13;12(4):e0175567. doi: 10.1371/journal.pone.0175567 (PMC5391077; doi:10.1371/journal.pone.0175567)
Supplement: S1 File — It contains the Ethical Approval Form and translation document. (ZIP) [file pone.0175567.s001.zip › Ethical Review/2016-9-10 English translation.doc]

The Ethics Committee approval of the First Affiliated Hospital of Chongqing Medical University

The Applicant: Yunsheng Ou

Application project title: Comparison between titanium mesh and autogenous iliac bone graft to restore vertebral height through posterior approach for the treatment of thoracic and lumbar spinal tuberculosis

The research programs and informed consents of this project were in line with the requirements of the basis ethic principle of medicine and the Declaration of Helsinki after the examination of the Ethics Committee. This project has scientific basis and will not result in unnecessary risk. It is approved by a predetermined implementation.

The Ethics Committee approval of the First Affiliated Hospital of Chongqing Medical University

August 15, 2016
