# Supplementary material for: Comparison between titanium mesh and autogenous iliac bone graft to restore vertebral height through posterior approach for the treatment of thoracic and lumbar spinal tuberculosis
Source: PLoS One. 2017 Apr 13;12(4):e0175567. doi: 10.1371/journal.pone.0175567 (PMC5391077; doi:10.1371/journal.pone.0175567)
Supplement: S2 File — It’s a informed consent form template for clinical trials. (ZIP) [file pone.0175567.s002.zip › Informed Consent Form Template for clinical trials.doc]

**知情同意书**

**研究背景介绍：**

尊敬的患者：

您好！

您将被邀请参加一项，由重庆医科大学附属第一医院骨科脊柱外科主持的研究。该研究旨在探讨单纯后路病灶清除、植骨融合、内固定术治疗胸腰椎结核的临床疗效，并比较钛笼与自体髂骨块植骨重建椎骨缺损的疗效差异而进行一项研究，通过回顾性分析我院骨科脊柱外科2011年1月至2013年12月行单纯后路病灶清除、植骨融合内固定治疗的胸腰椎脊柱结核患者的临床资料。由于您符合上述标准而被邀请加入此项研究。

本知情同意书提供给您一些信息以帮助您决定是否参加此项临床研究。您参加本项研究是自愿的。本次研究已通过本研究机构伦理审查委员会审查。如果你同意加入此项研究，请看下面同意书。

请您仔细阅读，如有任何疑问请向负责该项研究的研究者提出。

**研究目的：**

结核病是人类最古老的疾病之一。脊柱结核是骨关节结核的最常见感染部位，结核杆菌常感染椎间盘和椎体临近上下终板，破坏严重者将导致脊柱后凸畸形。手术已成为部分脊柱结核重要的治疗方式，结核病灶清除后所致骨质缺损往往需行结构重建，而重建脊柱所用植骨材料有较大争议，关于钛笼植骨和髂骨块植骨两种方式的临床疗效仍缺少足够证据。该文通过回顾性分析单纯后路病灶清除、钛笼与髂骨块植骨和内固定治疗胸腰椎结核的临床资料，旨在探讨钛笼与自体髂骨块植骨在胸腰椎结核单纯后路病灶清除术后脊柱重建的临床疗效与差异。

**研究过程和方法（简写）**：

回顾性分析了我院2011年1月到2013年1月行单纯后路病灶清除、植骨融合内固定治疗的胸腰椎脊柱结核患者临床资料，根据植骨方式和病椎节段分为单节段和多节段钛笼和髂骨植骨组。分别对比研究手术时间、术中出血量、VAS、ODI、ESR、CRP、Cobb 角和椎间高度矫正度及其丢失、神经功能恢复情况、植骨融合时间、并发症等。

**研究可能的受益**：

通过病历随访分析最终得出结论将有助于临床决策提供依据，理论性及实用性强，将使广大脊柱结核患者及参加试验患者受益。

**研究风险与不适**：

该研究为回顾性研究，无任何风险与不适。

**其他治疗干预方式：**

该研究非干预性试验。

**隐私问题：**

如果您决定参加本项研究，您参加试验及在试验中的个人资料均属保密。对于您来说，所有的信息将是保密的。该试验将以匿名方式登记，不会向其他第三方提供该资料数据。该项研究结果发表时，不会出现其个人信息，患者隐私将充分得到保护。

**费用和补偿：**

无任何其他费用及补偿，患者定期骨科脊柱外科门诊随访检查均自费。

**自由退出：**

作为受试者，您可随时了解与本研究有关的信息资料和研究进展，自愿决定（继续）参加还是不（继续）参加。参加后，您可以选择在任何时候通知研究者要求退出研究，您的数据将不纳入研究结果，您的任何医疗待遇与权益不会因此而受到影响。如果继续参加研究，会对您造成严重的伤害，研究者也将会中止研究的进行。

但在参加研究期间，请您提供有关自身病史和当前身体状况的真实情况；告诉研究医生自己在本次研究期间所出现的任何不适；告诉研究医生自己在最近是否曾参与其他研究，或目前正参与其他研究。如果因为您没有遵守研究计划，研究医师可以终止您继续参与本项研究。

**联系方式：**

如果您有与本研究有关的问题，或您在研究过程中发生了任何不适与损伤，或有关于本项研究参加者权益方面的问题，您可以与**课题组负责人欧云生医师（电话：18696669699）联系。**

**试验后利益分享：**

如果该研究得到肯定结果，可为临床决策提供理论依据，提高脊柱结核患者生活质量和预后。

**知情同意签字：**

我已经阅读了本知情同意书，并且我的医生欧云生医师已经将此次临床试验的目的、内容、风险和受益情况向我作了详细的解释说明，对我询问的所有问题也给予了解答，我对此项临床研究已经了解，我自愿参加本项研究。

受试者签名：_________________________

日期：______ _年________月________日

授权亲属签名：_________________________

日期：______ _年________月________日

研究者签名：_________________________

日期：______ _年________月________日
